# Analysis and Comparison of the Corrosive Behavior of Nickel-Based and Cobalt-Based Dental Alloys

**DOI:** 10.3390/ma14174949

**Published:** 2021-08-30

**Authors:** Carmen Marina Garcia-Falcon, Tomas Gil-Lopez, Amparo Verdu-Vazquez, Julia Claudia Mirza-Rosca

**Affiliations:** 1 Nanoscience and Nanomaterials, Department of Mechanical Engineering, University of Las Palmas de Gran Canaria, 35017 Las Palmas, Spain; carmen.garcia110@alu.ulpgc.es (C.M.G.-F.); julia.mirza@ulpgc.es (J.C.M.-R.); 2 Science and Engineering, Department of Building Technology, Madrid Polytechnic University, 28040 Madrid, Spain; amparo.verdu@upm.es

**Keywords:** metal alloys, NiCr, CoCr, electrochemical characterization, corrosion, Ringer solution

## Abstract

Nickel-based and cobalt-based metal alloys are frequently used in dentistry. The introduction of various elements in the alloy changes its characteristics, and a thorough study of each alloy should be completed to determine its appropriate corrosion resistance and biocompatibility in contact with physiological fluids. There are scarce investigations on these widely used dental alloys in Ringer solution, and findings in this research bring new experimental data and information. The present study evaluated and compared the corrosion behavior of six NiCr- and two CoCr-based dental materials in Ringer solution, using the following techniques: potentiostatic polarization curves (chronoamperometry), microstructural analysis, and EIS (electrochemical impedance spectroscopy). The results obtained in this investigation showed that in the NiCr-based specimens Ni4, Ni5, and Ni6 the stability of the passive layer was destroyed after polarization and a development and growth of stable pits was found in the microstructural analysis after electrochemical treatment. In terms of susceptibility to corrosion, two different groups of specimens were derived from this investigation. A first group which included the two CoCr (Co1 and Co2) and three of the six NiCr alloys studied (Ni1, Ni2, and Ni3). A second group with the other NiCr alloys investigated Ni4, Ni5, and Ni6.

## 1. Introduction

Nickel-based and cobalt-based metal alloys are frequently used in dentistry for prosthodontic restorations due to their advantageous characteristics [1,2,3,4,5,6,7,8]. Nickel in an alloy can cause allergic reactions and toxicity, according to some studies [9,10,11,12,13], but others report very different results and conclusions [14,15,16]. Furthermore, the introduction of chromium in their composition favors the stability of the alloy in order to be used as biomaterial [17,18]. According to several investigations, the chromium’s percentage found in the alloy is a decisive factor in the formation of the passive layer and the resistance to corrosion [19,20,21]. Cobalt-based alloys are frequently used biomaterials with applications in the dental and cardiac fields, as well as in orthopedic implants [22,23], due to the considerable wear and corrosion resistance properties given by this metallic element [24,25]. As dental materials, they are used for crowns with porcelain fused to metal [26,27,28,29,30], in fixed and removable dental prostheses [31,32,33,34,35], orthodontic wire leads [36,37], oral implants [38,39,40], and are very suitable in patients whose exposure to nickel might cause an allergic reaction [41,42].

The introduction of other elements in the alloy varies its characteristics. Aluminum, iron, copper, manganese, molybdenum, niobium, silicon, and tungsten can be found on different Ni- and Co-based alloys. As an example, it has been reported that the molybdenum content increases the corrosion resistance of the alloys [20,21] and their appropriateness to be used in the human body [19] as a biomaterial. Therefore, a thorough study of each alloy should be completed in order to determine its corrosion resistance and biocompatibility. Current research is focused on the investigation of materials to be used in the human body [43].

The alloys studied in this investigation, whether nickel- or cobalt-based, all had chromium in their composition, which makes them supposedly stable and safe to be used as dental alloys and are found in world markets for prosthodontic restorations. However, not all alloys have the same biocompatibility in contact with physiological fluids. The resistance to corrosion is the most important factor to be taken into account, because due to the corrosion process, elements are released into the oral cavity, causing problems that make biological safety difficult [44,45]. Certainly, materials in contact with human tissue must be non-toxic and not cause allergies or inflammations to be biocompatible [46].

Accordingly, in order to safely use these NiCr and CoCr alloys, a thorough analysis must be performed regarding their corrosion behavior. Electrochemical studies for a limited number of NiCr and CoCr alloys in artificial saliva medium were conducted by our group [47,48,49], and we are now investigating in simulated body fluid (Ringer solution). We have recently presented our study of CoCr alloys [50], but there are scarce investigations on these widely used materials in Ringer solution, and further studies are imperative to analyze and compare these alloys. Findings in this research bring new experimental data and information on these worldwide used NiCr and CoCr dental alloys in simulated body fluid. The present study evaluated and compared the corrosion behavior of six NiCr and two CoCr dental materials in Ringer solution, using microstructural analysis, potentiostatic polarization curves (chronoamperometry), and EIS techniques.

## 2. Materials and Methods

### 2.1. Materials, Specimens Preparation

Six NiCr and two CoCr dental alloys commercially used for prosthodontic restorations were studied: three manufactured in Germany, two in Romania, and three in the United States. The dental materials will hereinafter be referred to as specimens Ni1–6 and Co1,2.

The compositions of the eight investigated dental materials are shown in Table 1 and Table 2.

The specimens were cut to 1 cm^2^ size and each was inserted into an epoxy resin disk. Then, the samples were mechanically abraded using emery paper up to 2500 grit and polished with a 1 µm suspension of alumina. Before testing, the specimens used were cleaned completely in ethyl alcohol and deionized water.

The Ringer solution used as corrosion medium in this investigation had the following composition: NaCl—6.8 g/L, KCl—0.4 g/L, CaCl2—0.2 g/L, NaCO_3_H—1 g/L, glucose—1 g/L, MgSO_4_·7H_2_O—0.2 g/L and NaH_2_PO_4_·H_2_O—0.14 g/L.

### 2.2. Microstructural Characterization

To study the microstructure of the six NiCr- and two CoCr-based dental materials, a chemical reactant containing 10 mL HNO_3_, 30 mL HCl and 20 mL glycerine [51] and an PME 3-ADL microscope (Olympus, Tokyo, Japan), were utilized in the investigation. After electrochemical treatment, an analysis of the surface modifications of the NiCr and CoCr alloys using the microscope was conducted.

### 2.3. Electrochemical Measurements

The analysis was conducted in a three-electrode electrochemical cell, using as a reference electrode a saturated calomel electrode (or SCE), a platinum auxiliary electrode, and the sample as working electrode. A Princeton Applied Research (PAR, Oak Ridge, TN, USA) model 263A potentiostat, a lock-in amplifier 5210 (PAR, Oak Ridge, TN, USA), and a computer with Electrochemistry Power Suite software (PAR, Oak Ridge, TN, USA) were used.

#### 2.3.1. Potentiostatic Polarization Studies—Chronoamperometry

Chronoamperometry measurements were performed at a potential of 0.1 V/ESC on the working electrode. Using the electrochemical chronoamperometry technique, the induction time could be determined prior to the increase in the current density due to the breakdown of the passive layer. The current density variations of each of the different NiCr and CoCr dental alloys polarized at +100 mV/ESC in Ringer solution for 5 h were analyzed in this research. All tests were performed three times, and data acquisition and processing was performed with PowerCorr Princeton Applied Research software (PAR, Oak Ridge, TN, USA). After the potentiostatic polarization tests, the microstructures of the alloys’ surfaces were examined with the ADL microscope OLYMPUS PME3 (Olympus, Tokyo, Japan).

#### 2.3.2. EIS—Electrochemical Impedance Spectroscopy

EIS tests of the eight NiCr- and CoCr-based alloys were conducted for analysis and comparison of the corrosion resistance in Ringer solution following the potentiostatic polarization studies. The EIS spectra were recorded at the 100 mV/ESC potential after plotting the potentiostatic curves for 5 h.

Experimental EIS results were analyzed with ZSimpWin Princeton Applied Research software (PAR, Oak Ridge, TN, USA) to obtain the equivalent circuit (EC) where experimental data and simulated responses fitted well. Following each experiment, impedance data were displayed as Nyquist plots, Bode |Z|, and Bode phase diagrams. All tests were performed three times.

## 3. Results and Discussions

**Figure 1:**
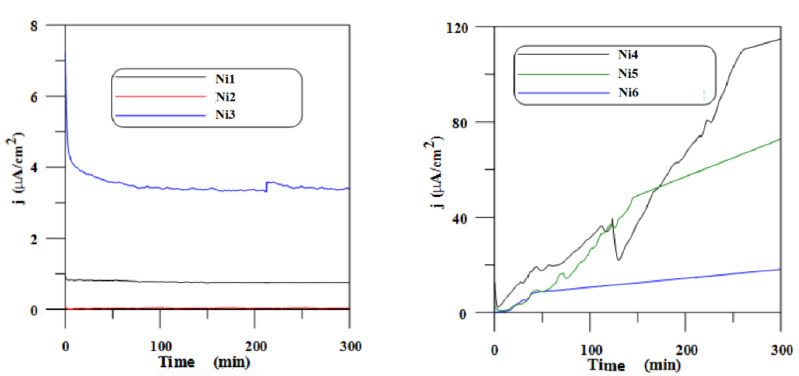
Potentiostatic polarization curves for specimens Ni1, Ni2, Ni3, Ni4, Ni5, and Ni6 in Ringer solution at a potential of 100 mV/ESC.

The potentiostatic polarization curves (chronoamperometry) for NiCr alloys in Ringer solution at a potential of 100 mV/ESC are shown in Figure 1.

In human organisms, pure titanium may be exposed to a maximum potential of about 450 or 550 mV/ESC [52]. For Co–Cr–Mo biomaterials, this information could not be found. Nevertheless, for physiological conditions in the human body, a metallic biomaterial’s potential value may fluctuate between −1.0 and 1.2 V, according to Black’s diagram of the potential pH [53].

In these conditions, it was found for some tested materials with very low potentials, that the stability of the passive layer in human organisms may be achieved at +100 mV/ESC. For this purpose, the potentiostatic polarization curves were plotted at a potential of +100 mV/ESC for 5 h, to prove the stability of the passive layer at this potential of +100 mV/ESC potentially achievable in the human body.

In the case of NiCr-based dental alloys, the polarization current of specimens Ni1, Ni2, and Ni3 at 100 mV/ESC in Ringer solution oscillated around 0.8 µA/cm^2^, 0.2 µA/cm^2^, and 3.8 µA/cm^2^, respectively. Specimens Ni4, Ni5, and Ni6 showed an increase in current density, higher in the cases of specimens Ni4 and Ni5.

**Figure 2:**
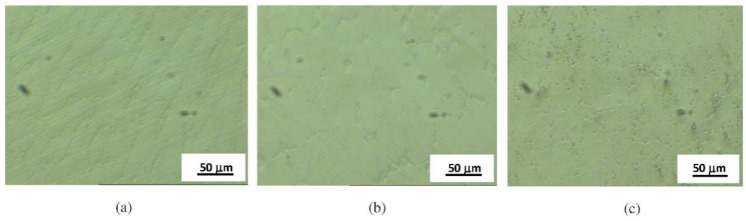
Microstructures after electrochemical treatment for (**a**) specimen Ni3, (**b**) specimen Ni1, and (**c**) specimen Ni2.

Microstructures of specimens Ni1, Ni2, and Ni3 after the 5 h potentiostatic treatment are shown in Figure 2. No degradation was observed in these specimens after the 5 h potentiostatic treatment.

**Figure 3:**
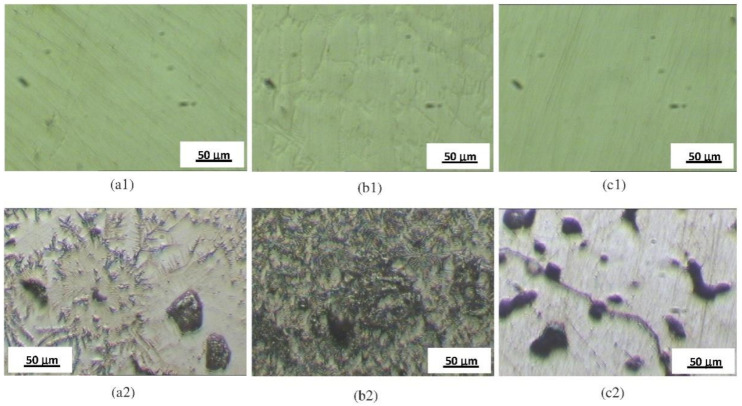
Microstructures before electrochemical treatments for (**a1**) specimen Ni5, (**b1**) specimen Ni4, (**c1**) specimen Ni6; after electrochemical treatments for (**a2**) specimen Ni5, (**b2**) specimen Ni4, and (**c2**) specimen Ni6.

For specimens Ni4, Ni5, and Ni6 the increase in current density is probably caused by the active anodic dissolution of the surface due to the film breakdown, with the formation and growth of stable pits. In Figure 3, microstructures of specimens Ni4, Ni5, and Ni6 before and after electrochemical treatments are presented.

**Figure 4:**
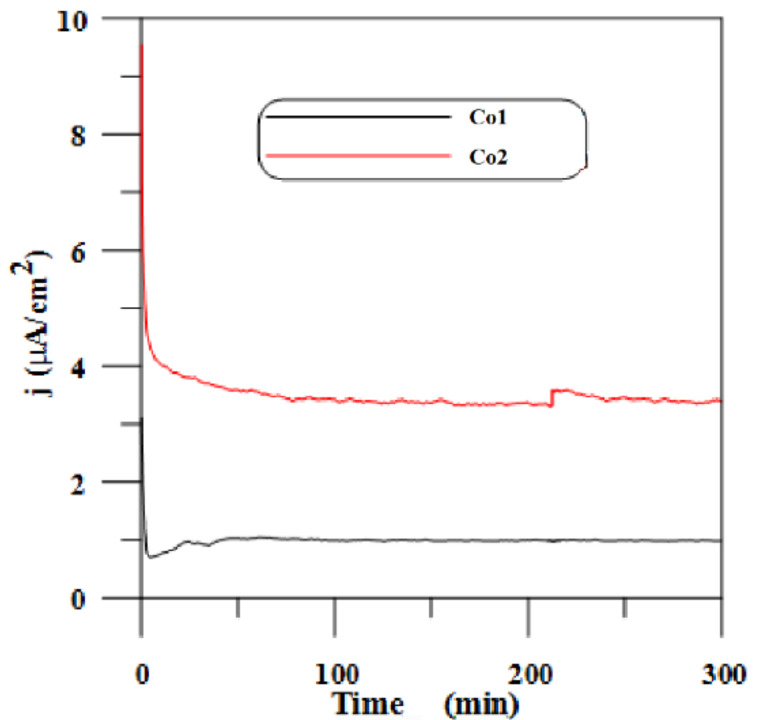
The potentiostatic polarization curves for specimens Co1 and Co2 in Ringer solution at a potential of 100 mV/ESC.

The potentiostatic polarization curves for CoCr alloys in Ringer solution at a potential of 100 mV/ESC are displayed in Figure 4.

The polarization current of specimens Co1 and Co2 at 100 mV/SCE in Ringer solution fluctuated around 1 µA/cm^2^ and 4 µA/cm^2^, respectively.

**Figure 5:**
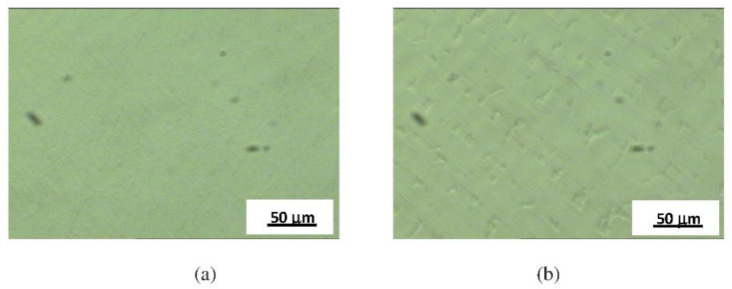
Microstructures after electrochemical treatments for (**a**) specimen Co2 and (**b**) specimen Co1.

Microstructures of specimens Co1 and Co2 after 5 h of potentiostatic treatment are shown in Figure 5. No degradation was observed in the CoCr-based dental alloys studied after the 5 h potentiostatic treatment.

**Figure 6:**
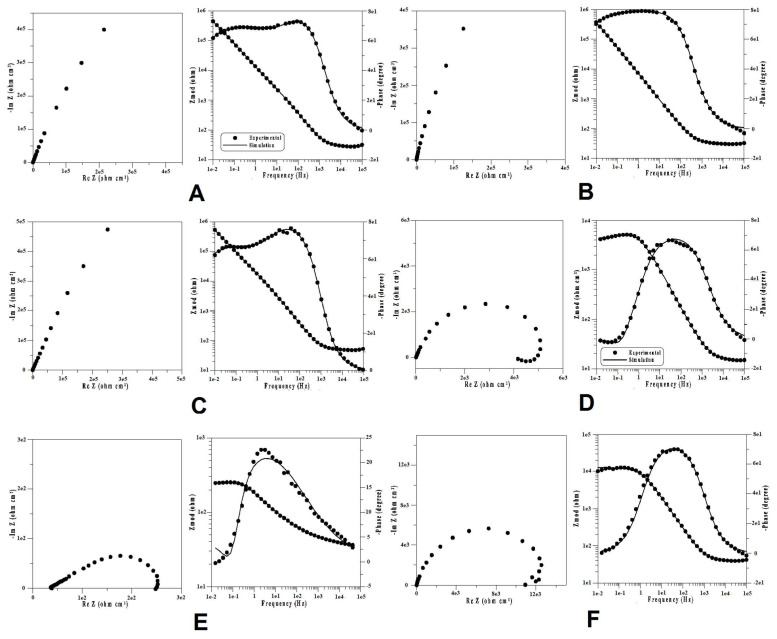
Bode spectra and Nyquist plot diagrams for NiCr-based dental alloys in Ringer solution at a potential of 100 mV/ESC. (**A**) Specimen Ni1. (**B**) Specimen Ni2. (**C**) Specimen Ni3. (**D**) Specimen Ni4. (**E**) Specimen Ni6. (**F**) Specimen Ni5.

After plotting the potentiostatic curves for 5 h, EIS spectra were recorded at the same potential of 100 mV/ESC. Representative results of Bode spectra and Nyquist plot diagrams for the NiCr-based dental alloys, polarized at 100 mV/ESC in Ringer solution, are shown in Figure 6.

**Figure 7:**
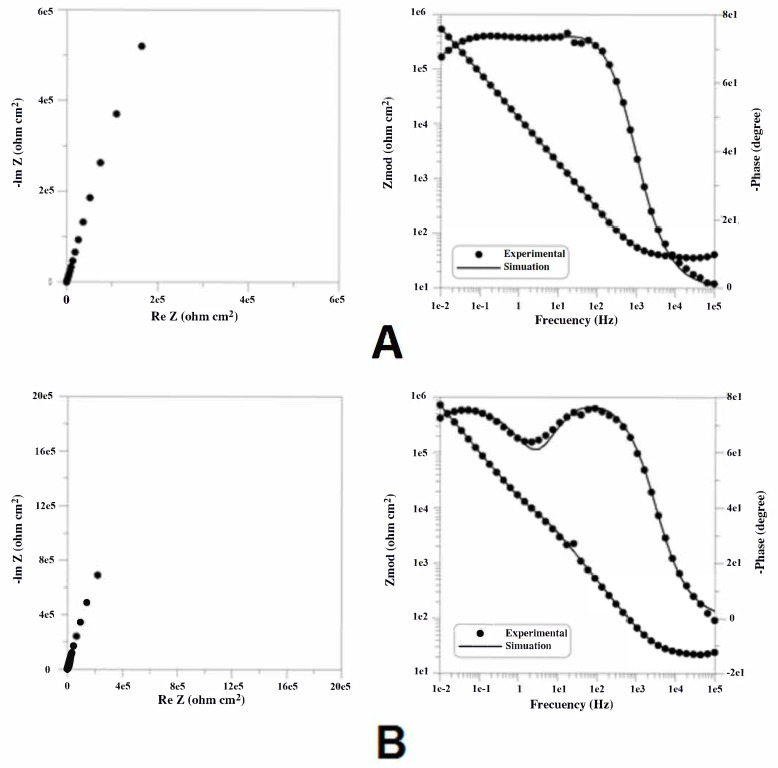
Bode spectra and Nyquist plot diagrams for CoCr-based dental alloys in Ringer solution at a potential of 100 mV/ESC. (**A**) Specimen Co1. (**B**) Specimen Co2.

Representative results of Bode spectra and Nyquist plot diagrams for the studied CoCr-based dental alloys, polarized at 100 mV/ESC in Ringer solution, are shown in Figure 7.

The experimental measurements are presented in the diagrams as distinct points, and the theoretical spectra that resulted from the equivalent circuit model used are displayed as lines.

The Nyquist spectrum showed that all alloys had a capacitive behavior with the immersion time in Ringer solution, except specimens Ni4, Ni5, and Ni6, which showed an inductive arc. In the electrochemical system, this arc can be associated with the process of metallic dissolution, showing values that are negative for the imaginary impedance [54].

An equivalent circuit, EC, gives the most notable corrosion indicators that can be applied to the substrate–electrolyte system and is formed by a group of different capacitors, resistances, and other circuit components. It is essential to have a proper model of the electrochemical reactions taking place at the electrodes to be able to interpret the system’s electrochemical behavior from EIS spectra. An EC representing an electrochemical cell displays impedance to a small sinusoidal excitation.

**Figure 8:**
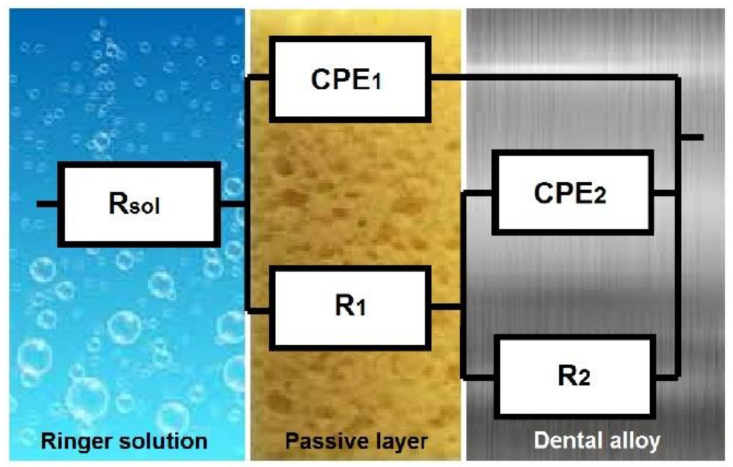
EC used to generate the simulated data for specimens Ni1, Ni2, Ni3, Co1, and Co2.

Starting with the easiest one, several models of electrical circuits were examined when analyzing the impedance data [28,55] for specimens Ni1, Ni2, Ni3, Co1, and Co2, with the best fit obtained for all the determinations using the EC presented in Figure 8.

In the model presented in Figure 8, the ohmic resistance of the electrolyte was designated R_sol_, the resistance of the passive film was designated R1, the passive film capacitance was represented as CPE_1_, the charge transfer resistance (R_ct_) was designated R_2_, and the double-layer capacitance was represented as CPE_2_. The EC model was very similar to that from M. Meticos-Hukovic et al. [56] for CoCr alloys dipped in Hank’s solution. As a result of the heterogeneous and thin oxide layer formed on the surface of the metallic alloys and the noticeable Bode plots’ deviations, it was necessary to substitute the “ideal” capacitance with a constant phase element (CPE), for which impedance is given by Z = (jω)−nY_0_, where j is an imaginary number (j^2^ = −1), ω is the angular frequency (rad·s^−1^), Y_0_ is the constant of CPE (Scm^−2^s^n^), n is the power number indicating the deviation from ideal behavior, n = α(π/2), and α is the constant phase angle of the CPE (rad).

The main parameters of the EC model for specimens Ni1, Ni2, Ni3, Co1, and Co2 are shown in Table 3. These parameters had the same meaning for all the alloys studied. The value around 10^−4^ from the χ^2^ or chi-squared distribution test proves that it was correct to use the constant phase element in the EC model, and also indicated a very good correspondence of fitted values and experimental data.

**Figure 9:**
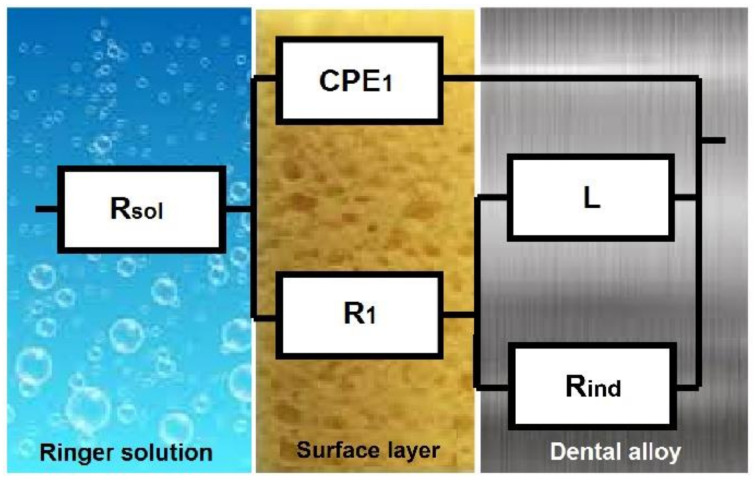
EC used to generate the simulated data for specimens Ni4, Ni5, and Ni6.

In the case of specimens Ni4, Ni5, and Ni6 polarized for 5 h in Ringer solution at a potential of 100 mV/ESC, the best simulations were performed using the equivalent circuit exhibited in Figure 9; the main parameters are shown in Table 4.

As previously stated, the value of around 10^−4^ from the χ^2^, or chi-squared distribution test, proved that it was correct to use the constant phase element in the EC model, and indicated an outstanding correspondence of fitted values and experimental data.

In the model exhibited in Figure 9, the ohmic resistance of the electrolyte was designated R_sol_, and the surface film resistance and capacitance were designated R_1_ and CPE_1_, respectively. It was found that the presence of an inductive process was characterized by a resistance R_ind_ and an inductance L, associated with an adsorption–desorption process that occurred in the formation of the surface film.

For this circuit, the total impedance was:

(1) $$Z_{eq}=R_{sol}+\frac{1}{jwC_{1}+\frac{1}{R_{1}+\frac{1}{\frac{1}{R_{ind}}+\frac{1}{jwL}}}}$$

After standard calculations, the following equation was obtained:

(2) $$Z_{eq}=R_{sol}+\frac{R-w^{2}RT+w^{2}AB}{{\left(1-w^{2}T\right)}^{2}+w^{2}R^{2}}+jw\frac{B-RA-w^{2}TB}{{\left(1-w^{2}T\right)}^{2}+w^{2}A^{2}}$$

where R = R_1_ + R_ind_, T = τ_1_ τ_2_, A = τ_1_ + τ_2_ + C_1_ R_ind_, B = τ_2_ R_1_, τ_1_ ≡ time constant of process at passive layer [s], and τ_2_ ≡ time constant of inductive process [s].

The equivalent circuit has a physical meaning associated with the passive layer itself, R_2_CPE_2_, and the passive layer/electrolyte interface, R_1_CPE_1_. The passive film was not destroyed by polarization at 100 mV/ESC for the two CoCr-based alloys (Co1 and Co2) or the NiCr-based alloys Ni1, Ni2, and Ni3. This fact was confirmed by potentiostatic polarization curves and surface microscopy after polarization.

From the data presented in Table 3, it was found that the stability of the materials was high at this potential due to the polarization resistance, which had high values compared to those obtained after one week of immersion in Ringer solution (greater than 10^5^ Ω cm^2^). The most stable alloy at the 100 mV/ESC potential was one based on CoCr, specimen Co2. Its polarization resistance of 10^6^ Ω cm^2^, according to different studies and the ASM Handbook [57,58,59,60], is characteristic of alloys with very high corrosion resistance.

The resultant parameters of the equivalent circuit, for the other three NiCr alloys, are presented in Table 4. Results showed that after polarization at 100 mV/ESC, the passive layer was destroyed (the inductance L is associated with the film dissolution).

When specimens Ni4, Ni5, and Ni6 were polarized at 100 mV/ESC, the passive layer developed on these alloys was considerably destroyed, and the impedance of the alloys was related to the R_ct_ or charge transfer resistance. As a result, there was no protective passive layer.

Additionally, it was observed that out of the three alloys, depending on the value of the polarization resistance (R_p_ = R_1_ + R_ind_), the highest stability was presented by specimen Ni5 and the lowest by specimen Ni6. A comparison with the polarization resistance values obtained after 7 days of immersion in Ringer solution revealed decreases of approximately 70-fold in the case of specimen Ni5, nearly 100-fold in the case of specimen Ni4, and about 500-fold for Specimen Ni6.

## 4. Conclusions

This investigation evaluated and compared the corrosive behavior of six NiCr- and two CoCr-based dental alloys in Ringer’s solution. Using potentiostatic polarization curves (chronoamperometry), microstructural analysis, and EIS, the following conclusions were derived:

1. The stability of the passive layer was not destroyed for the CoCr-based specimens Co1 and Co2, or the NiCr-based specimens Ni1, Ni2, and Ni3. This fact was confirmed by potentiostatic polarization curves and surface microscopy after polarization;
2. In the cases of specimens Ni4, Ni5, and Ni6, it was found that the passive layer was destroyed after polarization. Therefore, there was no longer a protective passive layer on these alloys;
3. Findings from the micrographs of the different NiCr and CoCr dental alloys studied after electrochemical treatments showed that there was no degradation for specimens Ni1, Ni2, Ni3, Co1, and Co2, but the development and growth of stable pits was discovered on the surfaces of specimens Ni4, Ni5, and Ni6;
4. According to the results obtained, in terms of susceptibility to corrosion from the spectral data, the NiCr and CoCr dental alloys were divided in two different groups. A first group which included the two CoCr (Co1 and Co2) and three of the six NiCr alloys studied (Ni1, Ni2, and Ni3), where the polarization resistance showed high values. In this group, the most stable alloy was specimen Co2, with a polarization resistance in the order of 10^6^ Ω cm^2^, characteristic of alloys highly resistant to corrosion. A second group with the other NiCr alloys investigated, Ni4, Ni5, and Ni6, where the passive layers were destroyed after polarization and the polarization resistance determinations were significantly lower than those exhibited by the first group. In this second group, specimen Ni5 had the highest stability and specimen Ni6 the lowest, based on polarization resistance values.

## Figures and Tables

**Table 1:** NiCr dental material composition.

Composition (in wt.%)	Specimens					
	Ni1	Ni2	Ni3	Ni4	Ni5	Ni6
Ni	60.1	60.8	63.4	72.1	64.9	53.4
Cr	24.3	23.9	23.2	20	17.9	14.4
Mo	10.1	8.8	3			
Fe	2.1	2.4	9	7.5		
Nb	1	3.8				
Si			1		1.8	1.5
Cu					9.9	9.5
Mn	2				3.6	19.4
Al					1.5	1.6

**Table 2:** CoCr dental material composition.

Composition (in wt.%)	Specimens	
	Co1	Co2
Co	63.5	63.4
Cr	27	29.0
Mo	5.5	5.2
Fe	2	
Ni	1	

**Table 3:** Main parameters of the EC used for specimens Ni1, Ni2, Ni3, Co1, and Co2.

Specimens	R_sol _ Ω cm^2^	R_1 _ Ω cm^2^	Y_01 _ Scm^−2^s^n^	n_1_	R_2 _ Ω cm^2^	Y_02 _ Scm^−2^s^n^	n_2_	χ^2^
Ni1	28	5 × 10^3^	8.9 × 10^−6^	0.83	5.5 × 10^5^	9.7 × 10^−6^	0.8	2 × 10^−4^
Ni2	35	3 × 10^3^	1.9 × 10^−5^	0.9	6.2 × 10^5^	1 × 10^−5^	0.88	4 × 10^−4^
Ni3	49	1.5 × 10^4^	8.4 × 10^−6^	0.89	5.9 × 10^5^	7.1 × 10^−6^	0.82	5 × 10^−4^
Co1	37	1.5 × 10^4^	7.8 × 10^−6^	0.9	9.1 × 10^5^	8.3 × 10^−6^	0.83	2 × 10^−4^
Co2	55	1.4 × 10^4^	6.1 × 10^−6^	0.9	1.2 × 10^6^	8.6 × 10^−6^	0.83	6 × 10^−4^

**Table 4:** Main parameters of the EC used for specimens Ni4, Ni5, and Ni6.

Specimens	R_sol _ Ω cm^2^	R_1 _ Ω cm^2^	Y_01 _ Scm^−2^s^n^	n_1_	R_ind_ Ω cm^2^	L Henri cm^2^	χ^2^
Ni4	20	4.5 × 10^3^	3.1 × 10^5^	0.8	1.7 × 10^3^	1.4 × 10^3^	8 × 10^−4^
Ni5	38	1.1 × 10^4^	1.5 × 10^−5^	0.84	9 × 10^2^	3 × 10^3^	6 × 10^−4^
Ni6	34	260	2.4 10^−3^	0.4	240	231	6 × 10^−4^

## Data Availability

The data presented in this study are available on request from the corresponding author.
